# Does cavity margin shaving reduce residual tumor and re‐excision rates? A systematic review

**DOI:** 10.1007/s00404-022-06512-5

**Published:** 2022-05-20

**Authors:** M. Fernandez‐Pacheco, O. Ortmann, A. Ignatov, E. C. Inwald

**Affiliations:** Department of Gynaecology and Obstetrics, University Medical Center Regensburg, Landshuter Straße 65, 93053 Regensburg, Germany

**Keywords:** Cavity shaving, Margin shaving, Cavity margin shaving, Lumpectomy, Breast‐conserving surgery/therapy, Breast cancer

## Abstract

**Purpose:**

Cavity shaving (CS) is a surgical technique used in the treatment of breast cancer (BC). It may reduce margin positivity in histologic assessment and consequently reduces re‐ excision rates in breast conserving surgery (BCS). The evidence for this assumption is described in the present review.

**Methods:**

A systematic review of relevant literature in English from January 1999 to April 2019 was conducted. The analysis included studies on CS and its effects on re‐excision rates and margin positivity. We searched PubMed databases for relevant publications. In total, 22 studies were included in the present review.

**Results:**

The benefit from CS on re‐excision rates and histologic margin positivity was variable. Out of 22 studies, 17 reported a reduction in both re‐excision rates and histologic margin positivity in margin shaved patients. Four studies could not find a significant reduction of second surgeries and residual tumor rates. One study suggested that CS after BCS was superior to single BCS only in subgroup analysis in IDC tumors.

**Conclusion:**

CS is a surgical technique that was shown to reduce re‐excision and margin positivity rates in most of the studies. Furthermore, it can be a useful tool to assess specimen margins and detect multifocality.

## Introduction

Breast cancer (BC) is the most frequent cancer in women worldwide. By now, BC is diagnosed at earlier stages due to wider use and higher sensitivity of BC screening. Thus, the majority of patients are suitable for breast conserving surgery (BCS). Provided that adjuvant radiotherapy is applied, patients with BCS have an equivalent survival rate compared to patients with radical mastectomy [1]. Although BCS is effective and more appropriate for most of the patients, it is associated with re‐excision rates up to 50% [2–4]. Many strategies of margin assessment have been introduced to reduce residual tumor on margins and, consequently, the need for re ‐excision. Cavity or margin shaving (CS) is a technique which consists in resection of the borders of the tumor bed after classical tumor excision.

Several studies demonstrated that CS could be an easy and effective procedure to decrease positive margin and re‐excision rates. Whether the classically performed excision of selective margins after radiologic or sonographic intraoperative imaging might be sufficient to improve residual tumor rates (R1/R0) remains unclear. The value of additional CS has been queried as multifocality might outweigh margin status in causing BCS failure. Moreover, excision of more tissue could result in poorer cosmetical outcome. Some authors propose that CS is a tool for assessing tumor margins more accurately and detecting multifocality. It is also claimed that CS may reduce re‐excision rates, which implies avoiding general anesthesia, surgical complications (e.g., haematoma, seroma, and scaring) and shortening of patient´s recovery time. Reducing re‐excision rates may also increase compliance and adjuvant therapy could be administer faster.

## Materials and methods

A systematic search of literature was performed using PubMed databases identifying studies published from January 1999 to April 2020. Studies were eligible for inclusion if they evaluated the role of CS in re‐excision rates in BCS and included at least 80 patients or more. The following groups of key words were used: (“cavity shaving” or “margin shaving” or “cavity margin shaving”) and (“lumpectomy” or “breast‐conserving surgery/therapy” or “breast cancer”). The language was limited to English publications.

### Study inclusion

The studies included vary from comparative studies, including randomized-controlled trials (RCTs) or non‐randomized studies (NRS), self‐control studies, which compared CS with standard lumpectomy in patients with BC undergoing BCS, reviews, and meta‐analyses. BC stages ranged from 0 to III. Different criteria for margin assessment such as “no ink on tumor” but also margin widths from 1 to 5 mm were accepted. However, margins assessed by imaging‐guide techniques were excluded. Only studies assessing the application of CS at the initial surgery were included. When multiple groups were analyzed in one study, the comparison between BCS plus CS and BCS alone was selected. Further evaluation of locoregional or distant recurrence rates was not examined in this review. The main questioning of this study were residual tumor rates and excision rates. Cosmetic outcomes and excised tissue volume were reported as non‐priority aspects.

### Data collection and quality assessment

The following information was extracted from the studies (Table 1): author, year of publication, study design, publishing journal, sample size (*n*), study periods, and outcomes of interest. To assess the quality of the studies, the Quality and Oxford Center of Evidence based Medicine (OCEBM) level of evidence (LOE) was used.

**Table 1:** Characteristics of the Included Studies

Study	Year of publication	Journal	Results summarized	Numbers of patients	Quality and Oxford center of evidence-based medicine (OCEBM) level of evidence (LOE)	Study period	Histological type of tumor	Margin width assessment
Systematic Review and Metaanalysis								
Wang et al. “Cavity Shaving plus Lumpectomy vs. Lumpectomy alone for Patients with BC undergoing BCS”	2017	PLOS one (Public Library of Science)	CS- groups in studies compared with BCS alone. CS group ↓pos. Margin rate and ↓RE (OR 0.41, 95% CI 0.32–0.53), *p* < 0.05 and OR: 0.42, 95% CI 0.3–0.59), *p* < 0.05	26 studies	Systematic Review and Meta-Analysis LoE 1a	1994–2016	Different	Different
Controlled randomized trials								
Chen et al. “Circumferential shaving of the Cavity in BCS: A randomized controlled trial”	2019	Annals of surgical Oncology	PMR postop 16,5 vs 7,8%, *p* = 0.073, RE 26,4 vs 23,3%, *p* = 0.64 in BCS vs BSC + CS CS no sign. ↓ PMR in BCS nor RE, benefit depends on vol of shaved tiss and breast size. DCIS only pred. factor for PMR	*N* = 181 (BCS alone 91, BCS + CS 90)	Randomized controlled trial LoE 2b	2016–2018	IDC ad DCIS	NA
Chapgar et al. “A randomized, controlled trial of Cavity Shave Margins in BC”	2015	N Engl J Med	2 groups, 19 vs 34% rate of pos. margins (with and without CS), *p* = 0.01, RE-rate 10 vs 21%, *p* = 0.02	*N* = 235, (119 CS–group, 116 non-CS- group	Randomized Controlled trial LoE 1B	2011–2013	IDC, DCIS and both	Within 1 mm
Prospective follow-up studies								
Malik et al. “Margin assessment by CS after BCS: analysis and follow-up of 543 patients”	1999	European Journal of Surgical Oncology	TBP in 37%. Total of 15% RE. 33.7% had R1. Pred factors of TBP grading, intraduct. component, young age, tumor size (*P* = 0.004, 0.038; 0.006; 0.007) CS sensitive to assess R0	*N* = 543	Follow-up study Prospective LoE 2b	1988–1995	IDC; ILC and DCIS	NA
Retrospective cohort studies								
Mukhtar et al. “BC and neg. margins in invasive lobular Carc.: the impact of OPS and SM in 358 Pat”	2018	Ann Surg Oncol	‐BCS ↓ R1 OR 0.4 (95% CI 0.21–0.79), CS OR 0.393, (95% CI 0.22–0.7) ‐pred factor for R1 breast and tumor size OR 1.81 (95% CI 1.583‐4.678), multifoc OR 2.721 (95% CI 1.583–4.678) -RE 25%	*N* = 358 (277 and 88 patients with single BCS and BCS + CS)	Retrospective cohort study LoE 3	1992–2017	ILC	No ink on tumor
Marudanayagam et al. “Effect of CS on Reoperation Rate Following BCS”	2008	The Breast Journal	RE 5.58 vs 12.5% *p* < 0.01. RE could have been avoided in 44 of 49 single BCS CS signif ↓ of RE	*N* = 786 (394 BCS + CS, 392 BCS)	Retrospective Cohort study LoE 3	2000–2005	IDC and ILC	Within 1 cm
Corsi et al. “CS reduces involved Margins and Reintervention without increasing costs in BCS: A propensity score-matched study”	2017	Annals of Surgical Oncology	Clear margins 98.3 vs 74.4% (*p* < 0.001), RE-rate 18.9 vs 1.9% (*p* < 0.001) in BCS + CS, OR for pos. final margin status 6.2 vs 5.46	*N* = 976 (BCS 164, BCS + CS 812)	Retrospective Cohort study LoE 3	2015–2017	IDC, DCIS, ILC	NA
Huston et al. “The influence of additional surgical margins of the total specimen volume excised and the reoperation rate after BCS”	2006	American Journal of Surgery	RE-rate was 38.7; 32.5 and 17.7%: BCS + CS 4–6 lowest RE but largest total vol specimen excis	*N* = 171 (BCS, BCS + CS 1–3 and BCS + CS 4–6, *N* = 49, 77.45)	Retrospective Cohort study LoE 3	2000–2006	IDC, DCIS and ILC	Within 2 mm
Kobbermann et al. “Impact of routine cavity shave margins on BC RE Rates”	2011	Ann Surg Oncol	RE total 31.9% (21.7 and 42% in BCS + CS and BCS), *p* = 0.015 -CS and EIC assoc with ↓ RE and R1 (*p* = 0.0003), (*p* = 0.005)	*N* = 138, (69 BCS + CS, 69 BCS only)	Retrospective cohort study LoE 3	2004–2009	IDC, DCIS and ILC	Within 2 mm
So et al. “The impact of pMRi and lumpectomy cavity shavings on RE-rate in pure DCIS-A single institution's experience”	2017	Journal of Surgical Oncology	CS and SSM (selective shaving margin 1–3) and BCS alone RE-rates of 24.5 vs 42.4%, *p* = 0.05, but no sign difference between single groups (24.8 vs 23.5 vs 42.4%; *p* = 0.12)	*N* = 176, (BCS + CS 109, 61.9%; BCS alone 33, 18.8%; BCS + SSM 34, 19.3%)	Retrospective, single-institution cohort study LoE 3	2010–2013	DCIS	Within 2 mm
Rizzo et al. “The effects of Additional Tumor Cavity Sampling at the Time of BCS on final status, volume of resection and pathologist workload”	2010	Ann Surg Oncol	↑neg. margins in BCS + CS (85.1 vs 57,2%, *p* < 0.05), in subgroup anal., only IDC showed this effect, not DCIS or IDC + assoc DCIS -No ↑in excised breast vol in CS	*N* = 320 (62,2% BCS and 37,8% BCS + CS)	Retrospective Cohort Study LoE 3	2004–2007	DCIS, IDC	Within 1 mm
Unzeitig et al. “Influence of surgical technique on mastectomy and RE-rates in BCT for Cancer”	2011	Int Journal of Surgical Oncology	RE-rate 43% (46.8 and 23.9% in each group), *p* = 0.0003. CS, DCIS%, tumor size and race preop pred factors for R1	*N* = 522, 455 BCS vs 67 BCS + CS	Retrospective Cohort Study LoE 3	NA	IDC, DCIS and ILC	Within 2 mm
Janes et al. “Systematic cavity shaves reduces close margins and RE-rates in BCS”	2006	The Breast	CS ↓close mar by 83% OR 0.17, 95% CI 0.08–0.48, *p* = 0.001 and ↓RE OR 0.26, 95% CI 0.09–0.74, *p* = 0.012. ↓tissue vol excised in CS	*N* = 217 (106 and 111 pat. in conventional BCS and BCS + CS)	Retrospective Cohort Study LoE 3	1999–2003	IDC, DCIS, ILC	Within 5 mm
Retrospective studies								
Pata et al. “Additional cavity shaving at the time of BCS enhances accuracy of margin status examination”	2016	Annals of surgical oncology	Tumor-free margins 90.7 vs 92.7 in BCS + CS; *P* = 0.69. RE 14.3 vs 10.6 in BCS + (*p* = 0.44). BCS + avoided RE in 5,6% Shaving does NOT ↓RE signif. (↓false pos margin, more accurate margin examination)	*N* = 298 (179 BCS + CS, 110 only BCS)	Retrospective, case-matched LoE 3	2013	ICD and DCIS	No ink on tmor
Hequet et al. “Surgical management modifications following systematic additional shaving of cavity margins in BCT”	2011	Annals of Surgical Oncology	In 25,3% RE avoided, in 6,1% multicentr. diagnosed despite inicial neg. margins Predictive factors for pos. margins ILC, tumor size, N1	*N* = 99	Retrospective LoE 3	2007–2008	IDC; ILC and DCIS	< 2 mm
Feron et al. “Interest in cavity shaving in BCT does not depend on lumpectomy technique”	2011	The Breast	RE avoided in 24% of cases by BCS + CS, in 6% diagnosis of multifocality. Usefulness of CS not related to volume of resection	*N* = 100	Retrospective LoE 3	2007	IDC, DCIS	< 3 mm
Cao et al. “Separate cavity margin sampling at the time of initial breast lumpectomy significantly reduces the need for re-excision”	2005	Am J. Surg Pathology	50% of pat. with pos. Lumpectomy margins (LM) were R1 in their CS but in 59% CS rendered final R0. signif ↓RE Young age, ↑pos. LM, grading EIC predic. factors for R1	*N* = 103	Retrospective LoE 3	2003–2004	ICD, DCIS	Within 2 mm
Tengher-Barna et al. “Prevalence and predictive factors for the detection of carcinoma in cavity margin performed at the time of breast lumpectomy”	2008	Modern Pathology	In 35% CS tumor found, in 20 cases RE avoided, in 13 done despite initial negativity US scan and tumor size predictive factors for R1	*N* = 107	Retrospective LoE 3	2003–2006	ICD, DCIS	Within 3 mm
Tang et al. “Lumpectomy specimen margins are not reliable in predicting residual disease in BCS”	2014	The American Journal of Surgery	Lumpectomy-and CS margins compared (35.5% LM pos, 24.4% CS pos) ‐no. of pos. Margins sign. Predictor (*p*: 0.008) ‐LM-status predicts R-status with 64,8% accuracy: 50.9% Sensibility; 69.5% specificity	*N* = 242	Retrospective LoE 3	2004–2006	IDC; DCIS	Within 2 mm
Jacobson et al. “Do additional shaved margins at the time of lumpectomy eliminate the need of RE?”	2008	The American Journal of Surgery	(all pats BCS + CS), CS avoided RE in 49%. No pred. factors for margin positivity	*N* = 125	Retrospective LoE 3	2002–2006	IDC, DCIS and ILC	Within 2 mm
Keskek et al. Factors predisposing to cavity margin positivity following conservation surgery for BC”	2004	EJSO	(All pats BCS + CS), in 60 cases RE avoided (initial margins R1, CS negative). Tumor size and ILC/DCIS pred factors for R1 (*p* < 0.001 and *p* = 0.043)	*N* = 303	Retrospective LoE 4	1997–2002	IDC, DCIS, ILC	Within 2 mm
Hewes et al. “Importance of routine cavity sampling in BCS”	2009	British Journal of Surgery	− 48% pos CS and neg marg − 10.8% neg marg, pos CS poor concordance pos margins and CS (32%)	*N* = 957 (all BCS + CS)	Retrospective LoE 3	1986–2007	IDC, DCIS, ILC	Within 1 mm

## Results

### Selection process

172 publications were identified after entering the keywords described above. Irrelevant records, which did not deal with the topics “re‐excision” and/or “cavity margin shaving”, were excluded. Studies in which CS was performed as a second surgery were also excluded. Finally, 22 publications were assessed for eligibility. All of them analyzed the effect of margin shaving on re‐excision rates. Of these, there was one systematic review and meta‐analysis and two randomized‐controlled trials. One study was a prospective follow‐up study.

Eighteen studies were retrospective: of these, nine were cohort studies and one was a case‐matched study. The 22 eligible studies were published between 1999 and 2019 with sample sizes from 99 to 976 patients. Patients with BC stages I–III with an indication for BCS were included. The distribution of histological types varied. Twelve studies included invasive ductal carcinoma (IDC), DCIS, IDC with extensive intraductal component, and invasive lobular carcinoma (ILC) [5–16]. Seven studies analyzed IDC and.

DCIS [17–23], only one IDC and ILC [24]. Two studies included only DCIS [25] or ILC [26]. The studies were assessed by the Quality and Oxford Center of Evidence-Based Medicine (OCEBM) level of evidence (LOE) score. The scores ranged from 1a to 3.

There was heterogeneity among the selected studies in definitions of margin shaving [27]. Three studies defined positive margins as 1 mm or less [8, 18, 22], and two studies took 3 mm as cut‐off [20, 23], whereas most of them (9 studies) took 2 mm as limit width [5, 7, 9, 11, 12, 15, 17, 25, 28]. One study defined 5 mm [10], another 1 cm [24], and three others “no ink on tumor” as the limit to margin positivity [21, 26, 29]. Thirteen studies compared the positive margin rate (PMR) between BCS and additional CS with the classic BCS with or without selective margin resection after radiologic or sonographic imaging assessment, whereas nine studies analyzed the effect of CS on margin positivity and re‐excision rates.

### Systematic review

A systematic review and meta‐analysis which included 26 studies by Wang et al. reported a reduction in margin positivity in the CS‐group vs the lumpectomy alone group (16.4% vs 31.9%) with an OR of 0.41 [95% CI 0.32–0.53, *p* < 0.05], which results in a OR reduction of 59% [16]. Also, re‐excision rates were lower in the CS‐group with an OR of 0.42 [95% CI 0.3–0.59, *p* < 0.05]. CS did not seem to increase excised tissue volume compared to BCS alone and it did not decrease locoregional or distant recurrence compared to BCS alone. Although high heterogeneity was present among studies, this claim remained significant for self‐control and comparative studies [OR 0.39; 95% CI 0.30–0.53]. This systematic review included 24 retrospective, non‐randomized studies and two randomized-controlled trials; one of them was the trial of Chapgar et al. which represented the most convincing and statistically powerful evidence of the benefit from CS vs BCS alone [18]. Even if Chapgar et al. could demonstrate this in univariate analysis, multivariate analysis could not show a superiority of CS over BCS alone.

### Randomized controlled trials

Chapgar et al. analyzed data from 235 patients in a two-armed randomized-controlled trial [18]. One group underwent simple BCS and the other BCS with CS. They found a difference in final histological margin positivity rates between the two groups (BCS with CS vs single BCS) after randomization of 19% and 34%, *p* = 0.01. The rate of margin positivity was very similar in both groups before randomization (56% and 34%, *p* = 0.69) and changed significantly after randomization for the group which obtained CS. 53% of 119 patients in the CS‐group had positive margins before randomization and had R0‐status after CS. In 12% of the 76 patients in the CS‐group with negative margins before randomization, further tumor was found due to multifocality. This questions the accuracy of margin status in predicting the existence of residual tumor. Chapgar et al. considered that there was a significant lower re‐excision rate among patients in the CS‐group (10%) compared to the single BCS‐group (12%) (*p* = 0.02).

They also found a higher risk for second and third re‐excision in the group which was not cavity shaved in comparison to the shaved group, but no significance could be reached for this observation (*p* = 0.09). As a second study end point, excised tissue volume and cosmetic results in both randomized groups were evaluated. Even if excised tissue volume was higher in the CS‐group, final cosmetic result after patient’s evaluation was in both groups. In summarize, CS was shown to halve margin positivity rates and re‐excision rates in comparison to standard BCS with or without further selective margin excision after intraoperative imaging or palpation. Extensive ductal carcinoma in situ (DCIS) and margin positivity were significantly associated with higher re‐excision rates.

This trial was conceived with intraoperative randomization after classical BCS with or without further selective margin excision after intraoperative imaging or palpation. Four different surgeons were in charge of the interventions. The number of intraoperatively excised margins before randomization was left to the surgeon’s criteria, which could have altered final results. Furthermore, cosmetic evaluation was left to patient´s subjective perception instead of an assessment by a multidisciplinary expert panel following strict criteria.

In the randomized-controlled trial of Chen et al., 181 patients were assigned in a shave and no‐shave group after BCS to investigate the effect of CS on margin positivity rates [19]. Both groups had a homogeneous distribution with 90 and 91 patients. There was no significant reduction in re ‐excision rate, with 26.4% vs 23.3% after BCS or BCS with CS (*p* = 0.64).

Postoperative PMR were not significantly different between the single BCS and the CS group (16.5% vs 7.8%, *p* = 0.073). In contrast, margin positivity was reduced by 15% in the trial of Chapgar et al. [18], whereas in the trial of Chen et al., it was reduced only by 4.3% [19]. CS did not significantly reduce margin positivity rate, but its effect was associated with the excised tissue volume and with the patient´s breast size. In the subgroup analysis, patients with C–E cup breasts had a tendency to benefit from CS. Patients with small breast size were less likely to benefit from CS. Apart from that, the presence of DCIS was the only predictive preoperative risk factor for PMR in both groups.

The study conducted by Chen et al. could only find significant correlation between higher volume of shaved tissue and big breast size and lower margin positivity re‐excision rates [19]. Thus, the fact that this study was performed with Chinese patients, who tend to have smaller breast volume and more breast density could imply a bias. Besides, when compared with the trial of Chapgar et al., the volume of tissue excised was smaller (12 cm ^3^ vs 36,1 cm^3^) and almost all tumors (*N* = 181, 90%) of the total patients cohort of Chen et al.´s study had palpable tumors, which could also imply a certain bias. Neither Chapgar et al. nor Chen et al. analyzed the role of CS on patients who underwent neoadjuvant chemotherapy (NACT). This could have been interesting, since many authors describe different shrinkage patterns after NACT.

### Prospective follow‐up studies

A publication by Malik et al. analyzed additional shavings in 543 patients who underwent BCS with CS [13]. Tumor bed positivity (TBP) was found in 37% of all patients, leading to the conclusion that CS might be sensitive to reach R0 status. 543 patients were retrospectively evaluated for margin positivity and re‐excision rates. 15% (*N* = 63) of all patients underwent re‐excision or mastectomy after first surgery. 33.7% (*N* = 28) of the patients with histologically assessed positive margins in the first BCS had again residual disease in their re‐excision or mastectomy specimens, whereas 65% (*N* = 56) of those patients undergoing a second surgery had no benefit regarding R0‐status. It may be assumed that for most of the patients, tumor was already excised by CS. Since there was no comparison in this study with patients only undergoing BCS and a clear benefit of CS cannot be determined. Furthermore, a low percentage of patients with lobular carcinoma and “special type” carcinoma were included, which was not taken into account when analyzing residual tumor status after CS. Predictive factors of margin positivity were tumor grading, extensive intraductal component (EIC), young age, and larger tumor size.

### Retrospective cohort studies

Mukhtar et al. performed a retrospective cross‐sectional analysis with multivariate model of a cohort of 358 prospectively collected women with invasive lobular carcinoma. The rate of margin positivity was 43% and the re‐excision rate was 25%, which seems to be consistent with the other studies. They compared simple BCS with BCS with oncoplastic techniques or CS for final residual tumor [26]. They calculated an odds ratio (OR) for CS of 0.393 [95% CI 0.22–0.7], indicating that CS or oncoplastic techniques were associated with significantly lower PMR, though only when adjusted for tumor size and multifocality. There was no significant benefit from CS over oncoplastic techniques, both reducing margin positivity in 60% (*p* = 0.008). Though, excised volume was much higher in oncoplastic techniques than with CS (93 vs 65 cm^3^). Predictive factors of margin positivity were tumor size [OR 1.81, 95% CI 1.583–4.678] and multifocality [OR 2,721, 95% CI 1.583‐4.678]. The weaknesses of this study were the potential patient and provider bias as well as surgeon‐dependent factors.

In the study of Marudanayagam et al., 786 patients were retrospectively analyzed in cohorts and divided into subgroups: 394 patients had a BCS with CS and 392 had BCS only [24].

Invasive ductal carcinoma and DCIS were included in the study. The rates of re‐excision were 5.58% (22 patients) in BCS with CS and of 12.5% (49 patients) in standard BCS (*p* < 0.01). Of these 49 patients who needed reoperation, 46 underwent re ‐excision and 44 patients h ad clear resection margins. I n 44 of 49 cases, reoperation was avoided because of CS. Of the 394 patients who obtained BCS with CS, in 52 cases, tumor was found in cavity excision; in 22 cases, re‐excision was necessary; in 30 cases, tumor could be resected in sano. This means that CS could be a useful tool for detecting multifocality and DCIS. A major point of criticism was that differences in re‐excision rates and margin positivity between surgeons were not taken into account. Multifocality as an independent predictive factor for residual tumor on margin was not analyzed.

In the retrospective cohort study by Corsi et al., 976 patients were evaluated [6]. Of these, 164 patients underwent simple BCS, whereas 812 patients had BCS with CS. Clear margins had no ink on tumor. They showed a difference of clear margin rates of 98.3% vs 74.4% in BCS with CS vs single BCS groups (*p* < 0.001). Also, re‐excision rates were significantly lower in the BCS with CS group (18.9% vs 1.9%, *p* < 0.001). OR for positive margin status was 6.2 (95% CI 2.85‐13.46; *p* < 0.001) without CS, while OR for re‐excision was 5.46 (95% CI 2.21–13.46, *p* < 0.001). Residual tumor was detected in 20% of patients who underwent BCS with CS, though, in 18.3%, R0‐status was achieved with CS. The re‐excision rates were 18.9% vs 1.9% in BCS and BCS with CS (*p* < 0.001). A significant reduction of R1‐disease and re‐operation rates was found in luminal A and luminal B such as in triple‐negative breast cancer. In contrast, in HER2‐positive tumors, no benefit from CS was achieved, which is in contrast to other studies. This retrospective cohort study included a relatively high sample volume of patients and the groups had a heterogeneous distribution: the group of BCS with CS included 812 patients, the group of simple BCS 164 patients. Apart from the lack of randomization, only pathologic features were considered in tumors instead of genetic profiling, e.g., besides, there were more patients with luminal than with triple-negative or HER2‐positive breast cancer.

In the retrospective study conducted by Huston et al., 171 selected patients were compared in three different groups: patients who underwent single BCS, BCS with selective CS in 1–3 directions and BCS with CS in 4–6 directions. BCS with CS where 4–6 cavities were shaved had the lowest reoperation rate but the largest excised volume specimen. This could possibly affect cosmesis. Re‐excision rates were considerably high with 38.7% in the study by Huston et al. [9]. They differed in rates of 17.7%, 32.5%, and 38.7% with CS in all directions, selective CS and BCS without CS. Only two surgeons performed all surgeries. Moreover, the three group samples (45, 49 and 77 patients) were relatively small. The main strength of this study is the comparison between different surgical approaches. Although more breast volume was taken in the group of BCS with CS in all directions, it remains unclear whether the effect of lower re‐excision and margin positivity rates is due to volume removal or due to selective CS.

A retrospective, case‐matched and single‐institution analysis by Kobbermann et al. described similar reoperation rates of totally 31.9% [12]. In the population of 138 patients, 69 received CS after BCS, while the remaining 69 did not. The re‐excision rates varied significantly among CS and the single BCS‐procedure with 21.7% and 42% (*p* = 0.015). Of 15 patients with CS in their first surgery who needed reoperation, 60% required reoperation for close margins, whereas 40% due to ink on tumor. This distribution changed among the 29 patients who needed reoperation in the group of simple surgery. 51.7% had reoperation because of close margins and 48.3% because of positive margins. In addition, patients who underwent CS were less likely to have residual tumor in re‐excision samples than patients who underwent a simple BCS (20 vs 31%). Summarizing, the rate of reoperation was significantly higher when no CS was performed [OR 9.2; 95% CI 2.8–30.5; *p* = 0.0003], as well as when larger extent of intraductal component was found in samples [OR 7.0; 95% CI 1.8–27.0; *p* = 0.005]. CS was well standardized (no more or less than six additional shaved margins of at least 1 cm thickness) in this study, meaning that authors tried to define CS consistently. Though, the small number in group samples (69 patients in each group) and the retrospective nature of this study has to be taken into account.

In the retrospective study of So et al., 176 women undergoing BCS for DCIS were analyzed [25]. Re‐excision rates were analyzed in subgroup analyses: those patients who underwent preoperative imaging assessment with resonance imaging (pMRI) and afterward BCS with CS or selective CS, BCS with CS (4–6 margins were taken) and BCS with selective CS (1–3 margins were taken) alone were analyzed. Furthermore, subgroup analysis was performed for different high-volume breast surgeons to compare re‐excision rates. The groups were heterogeneous in their sample volume (*N* = 33 vs. *N* = 109 vs. *N* = 34). The largest excised volume was in the group with pMRI (*N* = 33) and selective shave margins (SM) (*N* = 34). In this study, only patients with DCIS were included. So et al. [25] compared CS and selective CS with the classical procedure of single BCS on re‐excision rates and found no significant difference among the three subgroups: 24.8%, 23.5%, and 42.4% re‐excision rates, (*p* = 0.12). They were able to find a significant difference of 42.4% and 24.5% when comparing single BCS with further excisions (both groups, CS and selective re‐excision together), *p* = 0.05. Re‐excision rates were not lowered by the use of pMRI; thus, any kind of margin shaving (CS or selective CS), the size, and grading of DCIS were associated with lower re‐excision rates. In bivariate analysis, surgeon-specific practice was demonstrated to be associated with histologic margin positivity. There was high heterogeneity among the three different surgeons regarding re‐excision rates (from 14.6 to 40.5%, *p* = 0.02). Procedures performed by surgeon B (OR 3.23, CI 1.04‐9.99; *p* = 0.04) and C (OR 3.57, CI 1.04‐12.33, *p* = 0.04) were associated with higher re‐ excision rates than those performed by surgeon A. The most important point of criticism is the small sample size, especially for subgroup analysis. In addition, the definition of “high‐volume breast surgeons” was the treatment of more than 10 patients per year, which does not represent a “high‐volume”.

Rizzo et al. also conducted a retrospective analysis of 320 patients who underwent BCS or BCS with CS [22]. Of all patients, 62.2% underwent BCS and 37.8% had BCS with additional CS. They found a higher rate of negative margins in BCS with CS compared to single BCS (85.1% vs 57.2%, *p* < 0.05) and a lower re‐excision rate. In subgroup analyses, this effect was only observed for invasive ductal carcinoma (91% vs 62.1%, *p* < 0.001), but not for DCIS or invasive ductal carcinoma with associated DCIS. Patients with DCIS only showed no difference in rates of negative margins when comparing BCS with BCS plus CS (51.1% and 69.7% of negative margin rate). In patients with invasive ductal carcinoma, there was a higher proportion of residual tumor on margins regardless of the surgical approach (42.9% vs 30.3%, *p* < 0.007). Only 6.8% of patients with invasive ductal carcinoma needed re‐excision after BCS plus CS while 24.5% of patients single BCS needed reoperation. No increase in excised breast volume could be found in the CS‐group. This study showed a homogeneous distribution in subgroups, since 37.8% of patients had DCIS, 32.3% invasive ductal carcinoma and 30% invasive ductal carcinoma with adjacent DCIS. Though, no subgroup analysis was performed regarding re‐excision rates. 80% of the study population was African American, which could be a considerable bias.

The highest re‐excision rates were reported by Unzeitig et al. with 43% [15]. In this retrospective, single‐institution cohort study, 522 patients were included. 455 of the patients had single BCS; 67 had BCS followed by CS. Considerable differences in re‐excision rates between single BCS and CS was found: 46,8% and 23.9% in each group (*p* = 0.0003). Re‐ excisions after CS were rather due to close margins (75%) than to residual tumor on margins. Residual tumor on margins were more common in the single BCS group, samples revealing residual tumor in 44.6% of re‐excisions. In the CS group, there were no patients who needed more than two surgeries (re‐excision or mastectomy), whereas in the single BCS group, 10.1% of patients had more than two surgeries to achieve R0‐status. However, there were no data regarding the change in surgical approach from simple re‐excision to mastectomy.

Factors which were associated with higher probability of re‐excision were no CS on BCS, DCIS, tumor size and race. According to this study, re‐excision rate could be lowered with CS from 46.8% before CS was introduced to 22.9%. One main point of criticism is the small sample of patients who underwent BCS with CS compared with those who obtained single BCS. This large difference in re‐excision rates between patients who underwent single BCS and BCS with CS found by Unzeitig et al. correlated with the findings described by Janes et al. with a reduction of re‐excision rates by CS [OR 0.26, 95% CI 0.09‐0.74, *p* = 0.012] [10].

Janes et al. recorded data from 217 patients undergoing BCS [10]. 111 patients (51.16%) with BCS and CS were compared with 106 patients (48.84%) with standard BCS with selective margin shaving according to radiologic or sonographic intraoperative assessment. Before CS or selective margin shaving, in the first group of patients receiving CS; 51.4% had residual tumor on margins before CS. In the group of standard BCS with selective margin shaving, 36.8% had close margins. A reduction by CS of both positive margin and re ‐excision rates was described. Re‐excision was avoided in 15 (7.3%) vs 8 (3,9%) cases. Despite of that, R1‐status was reduced by 83%, [OR 0.17, 95% CI 0.08–0.48, *p* = 0.001] by CS. CS was able to reduce re ‐excision rates in multivariate analysis [OR 0.26; 95% CI 0.09–0.74, *p* = 0.012].

Furthermore, Janes et al. were the only investigators finding that fewer tissue volume was excised in the group of CS compared to standard BCS [10].

Pata et al. conducted a retrospective case‐matched study of 298 women [21]. 179 of the patients received BCS with CS and 119 received simple BCS. The “no ink on tumor” policy for R0‐status was adopted. The difference was not statistically significant. However, if a wider margin policy (> 2 mm width from tumor) had been adopted, only 53.8% of patients would have negative margins in the group of simple BCS compared to 80.5% in the shaved group. This difference would have been indeed statistically significant (*p* < 0.0001). They reported tumor‐free margins in 90.7% in BCS vs 92.7% in BCS with CS (*p* = 0.69). No statistically significant difference could be seen between both groups regarding the removed volume.

Hequet et al. also described a reduction in re‐excision rates after CS in a retrospective study which included 99 patients who underwent BCS and systematic CS [7]. Carcinoma could still be found in CS‐margins of 29.3% (*N* = 29 patients), in 23 cases after initially negative margins in lumpectomy specimen, and in 6 cases after initially negative lumpectomy margins.

Thus, they claimed that CS might be useful in the diagnosis of multifocality. Of all patients with initially positive lumpectomy margins, 25.3% (*N* = 25 patients) had a negative cavity shaving histology. In these cases, false-positive results were avoided due to CS. Re‐excision was avoided in 25% of the patients who underwent BCS with CS, as they had positive lumpectomy margins but negative CS margins.

Significant association with carcinoma detection in cavity shaving histology could be found in patients with invasive lobular carcinomas, wider carcinoma diameter, positive lymph‐node status, and positive margins in wide local excision. No predictive risk factors for positive margins could be found and there was no group of patients that benefit from margin shaving. CS was performed in four directions in this study, and excision in ventral and dorsal direction was not counted, since the muscular fascia and part of the subcutis were excised. Though, surgery was performed by different surgeons and volume or width of the additional margins taken were not specified. This study analyzed only a relatively small number of patients (*N* = 99) retrospectively and could therefore not analyze whether there was an interpersonal surgeon‐dependent effect on positive margin status or not.

The reduction of re‐excision rates after CS claimed by Pata et al. was also not statistically significant with rates of 14.3% vs 10.6% in BCS with CS (*p* = 0.44) [21]. Though, CS avoided re‐ excision in 5.6%, which correlates with the findings of Hequet et al., in which re‐excision was avoided even in 25.3% of the cases [7].

In multivariate analysis, distance of tumor from initial lumpectomy margins, multifocality, receptor status, and tumor size were related to margin positivity in CS as predictive factors. Concluding, CS was shown to have no prognostic improvement regarding the “no ink on tumor” policy. Though, it provides wider clear margins, reinforces complete tumor removal (R0‐status) and helps overcoming false-positive margins with no wider volume removal.

Feron et al. examined 100 patients who underwent BCS and CS. Final histological results clearly showed residual tumor in 25% of cases, whereas R0‐status was obtained in 67% of the cases [20]. Initial lumpectomy margins were positive in 44 (44%) patients; 27 of them had residual tumor in CS margins on top. In 5 (6%) of the 52 patients who had negative lumpectomy margins, CS‐tissue was positive, i.e., multifocal disease was diagnosed. According to analysis of CS‐ tissue, re‐excision was avoided in 24% of the patients due to CS. Furthermore, diagnosis of multicentricity was possible in 6% of cases. No correlation between CS and the volume of excised breast tissue was found. However, usefulness of cavity shaving was not related to its resection volume. No preoperative characteristic was associated with positive lumpectomy margin or positive cavity shaving. One weakness of this study was the missing control group with patients who did not undergo systematic CS.

The study of Cao et al. analyzed 126 BCS histopathologic margins regarding their respective CS margins [17]. It showed that in 59% of the cases, margin shaving rendered final R0. 126 patients with IDC or DCIS or both who underwent BCS with CS were examined for margin positivity and other histological parameters. 50% of patients with residual tumor in initial lumpectomy margins had residual tumor in the CS‐margins. In 10.3% of the CS margins with initially negative BCS margins, carcinoma was still found, i.e., multifocal disease was diagnosed. Young age, elevated number of positive lumpectomy margins, tumor grading, and extensive intraductal component were significant predictive factors for final histological margin positivity [17]. This study only compared the histopathologic R‐status in associated margins. Different approaches from different surgeons were not taken into account. The sample of 126 patients was probably too small to be significant for subgroup analysis, since DCIS, invasive ductal carcinoma, and carcinoma with EIC were compared.

Tengher‐Barna et al. selected 107 patients with BCS with CS and analyzed the reoperation rates and the close margin rate [23]. Several demographic and tumor‐dependent factors were analyzed to find a tool to predict probability of residual tumor on margin and reoperation. They described tumor findings in 35% (*N* = 38 patients) of cavity shaved margins in their retrospective study. Of them, 33 patients underwent re ‐excision. When initial margins were positive and CS margins negative, in 20 of totally 107 cases (18.7%), re‐excision was avoided due to careful examination of CS margins. In 13 cases (12.2%), re‐excision was done despite initial margin negativity of tumor bed, rendering again a useful tool in diagnosis of multifocality. Of all analyzed factors, tumor size and non‐menopausal status were the only significant independent predictive factor for margin positivity.

Tang et al. analyzed pathology reports of 242 patients with invasive ductal carcinoma, DCIS and invasive lobular and tubular carcinoma and compared positivity of initial lumpectomy margins and its concordance to CS margins [14]. They found rates of 35.5% and 24.4% of margin positivity among patients and analyzed their predictive value. The number of positive cavity margins was a significant predictor for R1‐status (*p* = 0.008), whereas lumpectomy margins predicted R1‐status with 64,8% accuracy. This predictor had a sensitivity of 50.9% and a specificity of 69.5%. This study correlated with the findings of Heiss et al. [30]. CS margins were considered individually instead of all 6 margins together. When one margin of single BCS was involved by tumor, CS margin was negative in 65% of the cases. Vice versa, when one BCS border was negative, tumor was still found and CS margins in 18.6% of the cases. Both rates were higher than in other studies. This poor concordance of margin status between BCS borders and CS margins was the reason for recommendation of CS by the authors. Though, the election of the method of BCS (whether CS or not) was left to the surgeon´s discretion, as well as the thickness of shaved tissue.

Jacobson et al. performed a retrospective study with 125 patients who had undergone BCS with CS. In 66% (*N* = 83) of all tumor specimens residual tumor was found [5]. However, 34% (*N* = 42) of specimens were free of tumor. For 48% of patients, re‐excision was avoided due to CS. In 50% (*N* = 63) of the cases, the technique of CS did not change therapy management, since in 33% (*N* = 41), initial margins were already negative, and in 18% (*N* = 22), margins remained positive even after CS. According to this study, re‐excision rates tend to be rather high in the analyzed institution, since 66% (*N* = 83) of specimens had residual tumor after surgery. No control group with single BCS without CS was included for comparison.

Furthermore, no surgeon‐dependent factors (surgeon´s experience, surgeon’s number, etc.) were analyzed.

A retrospective, single‐institution study conducted by Keskek et al. analyzed 301 patients with 303 breast cancers [11]. Of these, 258 (85.2%) had no further surgeries, whereas 43 patients (14.8%) required secondary mastectomy. CS status was correlated to initial lumpectomy border. 170 (56.1%) of 303 cancers had both negative lumpectomy and CS borders, whereas in 13 cases (4.3%), only CS was positive, and in 60 (19.8%) cases, both initial borders and CS were positive. In conclusion, 73 patients (24.1%) had positive CS findings, which lead to more accurate diagnosis of multifocality. Due to CS, in 60 cases (19.8%), re‐ excision was avoided. Both large tumor size (*p* < 0.001) and the existence of ILC or DCIS (*p* = 0.043) were significant predictors for residual tumor on CS. An important bias of this study was that all surgeries were performed by one surgeon. There was also no comparison with additional CS and single BCS performed without additional shaving.

In the Breast Unit in Willesborough in the UK, a retrospective study by Hewes et al. gathered 957 patients over 21 years [8]. The initial margins of these patients after BCS and histology of cavity shave was compared. Patients were divided into four groups according to resection margin and CS: patients with positive margins and negative CS, patients with negative margins and positive CS, patients with both positive margins and CS, and patients with both negative margins and CS. Regarding demographic characteristics, patients in the CS ‐positive group were younger than in other groups. Of the 171 patients with positive CS, 48% had negative initial margins. Of the 761 patients with initial negative resection margins, 10.8% had positive CS. There was poor concordance between positivity of both initial margins and CS with only 32%. According to Hewes et al., there was only poor concordance between positivity of initial resection margins and CS [8]. Histology of the second surgery showed a higher percentage of DCIS in the CS‐positive group. Of the *N* = 171 patients with a positive CS, 82 (48%) had final R0. of the 761 patients with R0. 82 (10.8%) had a positive CS. There was poor concordance between positivity of resection margins and CS. Summarizing, CS is an important tool detecting residual and multifocal tumor, since margin and cavity positivity are often not concordant. A main point of criticism of this study is the lacking analysis of interpersonal differences in re‐excision rates between surgeons.

## Discussion

CS has been claimed to reduce secondary surgery caused by positive margins after BCS. The addition of CS differs from classical BCS in extent and width. Several sampling techniques have been described before. In contrast, CS is a relatively consistent method, with complete resection of the surface and sufficient width of the cavity wall. This review suggested that additional CS had a lower PMR than BCS alone.

According to the randomized-controlled trial of Chapgar et al. after randomization, there was a significant reduction of histologic margin positivity in BCS with CS compared to BCS alone (*p* = 0.01) [18]. Re‐excision rates also decreased by CS significantly (*p* = 0.02). CS was associated with a 59% OR reduction in the tumor‐involved margin and re‐excision rates [16]. This association was reinforced by Chapgar et al. who claimed that CS is supposed to reduce the rate of margin positivity in nearly 50% of the patients and more than halve re‐excision rates [18], although this number was not as high in other studies, i.e., Hequet et al. [7]. In another randomized-controlled trial performed by Chen et al., no significant reduction by CS was described neither in re‐excision rates (*p* = 0.65) nor in margin positivity (*p* = 0.07) [19]. This contrasts with the other big randomized-controlled trial by Chapgar et al. which can be due to different patient’s characteristics (i.e., smaller breast volumes in Asian population).

Whether CS is necessary remains unclear in the study conducted by So et al. in which no significance could be reached when comparing CS and selective shaving margins [25]. Chen et al. and Pata et al. did not find a significant reduction in reoperation after CS [19, 21].

Though, Pata claimed CS avoided re‐excision in some cases, reduced false-positive margin status and contributed to a more accurate margin examination, i.e., for multifocality. These findings correlated with the study of Hequet et al. in which multifocality was diagnosed in 6.1% despite initial negative margins [7]. Feron et al. showed that re‐excision was avoided in 24% of the patients due to CS and diagnosis of multicentricity was possible in 6% of cases [20]. Feron et al. found no correlation between CS and the volumes of excision of breast tissue.

Of all 18 retrospective studies, five found a significant reduction in margin positivity rates and, therefore, re‐excision rates, whereas in 13 studies, only a tendency for CS to reduce residual tumor or avoid re‐excision was seen. Summarizing, the majority of studies did not find statistical significance for CS to be a valid technique in reducing re‐excision rates.

Some studies described effects of CS only in certain subgroups, such as in invasive ductal carcinoma [22], multifocal tumors [24, 26], luminal A, B or triple-negative tumors [6], and lobular carcinoma [7, 11]. Even the distribution in subgroups among studies showed high heterogeneity. CS was not even found to be a useful tool in reducing re‐excision rates in subgroups, i.e., ILC or DCIS, which have always been claimed to have higher risk for re‐excision [30].

Out of 22 studies, 17 reported a reduction in both re‐excision rates and histologic margin positivity in margin shaved patients (37, 5, 22, 25, 23, 14, 18, 30. 35, 15, 11, 9, 3, 33, 32, 2, 17, 13). Four studies could not find a significant reduction of second surgeries and residual tumor rates [6, 19, 21, 25]. One study suggested that CS after BCS was significantly superior to single BCS only in subgroup analysis in IDC tumors [22]. This heterogeneity in results can be due to the lack of consistent definition of volume taken in CS after BCS, the different cohort samples, and the heterogeneity of subgroups (differences in histologic types, epidemiological characteristics in different populations, etc.). The differences were largest regarding institution’s characteristics, i.e., in number of surgeons involved, expertise of surgeons and different tumor assessment (mammographic, sonographic, or none).

Several of the 22 identified studies analyzed predictive risk factors for margin positivity or residual tumor which could predict risk for re‐excision. Kobbermann et al. found that CS and extensive intraductal component were significant risk factors: *p* = 0.0003 and *p* = 0.005 [12]. Unzeitig et al. described CS, percentage of DCIS, tumor size, and race as predictive factors for R1. This correlated with the findings of Thomas et al. [31]. Keskek et al. identified tumor size, invasive lobular carcinoma, and DCIS as significant risk factors [11], whereas others did not find predictive factors [5]. In contrast to Sioshansi’s findings [32], triple-negative tumors were not more likely having residual tumor than other subtypes.

This systematic review has several limitations. As retrospective studies or non‐randomized studies were included, recall bias and selection bias were unavoidable. The definition of positive of shaved margins was not consistent, and the standard of positivity varied between studies with respect to the distance from the cut edge. The width of the cavity shave margins and their orientation for histologic examination were in all studies according to the surgeon's decision and there was no standardization of the excised volume of CS. The surgeon‐based changes in re‐excision and margin positivity rates should be also considered after Valero et al. [33]. Another possible bias is that lumpectomy specimens with narrower margins were more likely to have residual disease in two or more margins shaved. In addition, BCS with selective excision of margins in dependence of intraoperative imaging techniques is a technique that has not been compared systematically with CS and could be also valid reducing re‐excision rates*.*

We conclude that CS is a surgical technique that may reduce re‐excision rates. However, there is limited high-quality evidence that supports general use of this method in BCS. Therefore, more prospective randomized-controlled trials are needed.
